# Lamina Cribrosa Curvature in Healthy Korean Eyes

**DOI:** 10.1038/s41598-018-38331-7

**Published:** 2019-02-11

**Authors:** Seung Hyen Lee, Tae-Woo Kim, Eun Ji Lee, Michaël J. A. Girard, Jean Martial Mari

**Affiliations:** 1 Department of Ophthalmology, Bundang Jesaeng General Hospital, Daejin Medical Center, Seongnam, Korea; 2 Department of Ophthalmology, Seoul National University College of Medicine, Seoul National University Bundang Hospital, Seongnam, Korea; 3 Department of Biomedical Engineering, National University of Singapore, Singapore, Singapore; 4 Singapore Eye Research Institute, Singapore National Eye Centre, Singapore, Singapore; 5 Université de la Polynésie française, Tahiti, French Polynesia

## Abstract

Given that posterior bowing of the lamina cribrosa (LC) is a principle event in the development of glaucomatous damage, assessment of the LC morphology may have clinical utility in diagnosing and managing glaucoma patients. LC curvature has been suggested as an index to evaluate the LC morphology. To apply LC morphology in clinical practice, it is necessary to know normal profiles of LC curvature in healthy population. This study was performed to investigate the characteristics of LC curvature in healthy eyes using enhanced depth imaging spectral-domain optical coherence tomography in a total of 250 eyes of 125 healthy Korean subjects. The lamina cribrosa curvature index (LCCI) values at seven locations spaced equidistantly across the vertical optic disc diameter were measured on serial horizontal B-scan images. The mean value of the seven measurements was defined as the average LCCI. The average LCCI was 7.46 ± 1.22 (range, 4.29–10.48) and did not differ significantly between the right and left eyes. There was a strong inter-eye correlation within subjects. LCCI was significantly larger in eyes with shorter axial length (*P* < 0.001). The observed range of LCCI in healthy subjects may be used as a reference for evaluating LC curvature in glaucomatous eyes.

## Introduction

The lamina cribrosa (LC) is considered the main supportive component of the optic nerve head (ONH)^1^, and also the putative primary site of axonal injury in glaucoma. Tensile strain within the LC can induce shearing stress on the axons passing through the laminar pores^2,3^. In addition, deformation and compression of the LC may promote optic nerve ischemia, because strain within the LC can induce possible occlusion of the laminar capillaries^4,5^. Thus, imaging and characterization of the LC morphology may not only expand our understanding of the pathogenesis of glaucomatous damage but help in developing better strategies in diagnosing and managing glaucoma.

Previous studies have attempted to characterize LC morphology using a measurable parameter^6^, the most frequent being LC depth measured from Bruch’s membrane opening (BMO) level (LCD__BMO_)^7–10^. Although baseline (innate) LC depth is unknown, large LC depth may be considered a surrogate indicator of large posterior LC deformation. However, the evaluation of posterior LC deformation using LCD__BMO_ may provide a biased assessment because the measurement is influenced by choroidal thickness, which is not associated with posterior LC deformation. Previously, our group proposed that curvature of the LC, evaluated using the lamina curvature index (LCCI), may be superior to LC depth as a parameter relevant to ONH biomechanics^11^. Unlike LCD__BMO,_ LC curvature is not affected by choroidal thickness.

To apply any index to characterize LC morphology, it is essential to determine this index in a healthy population. Such knowledge should provide a basis for the recognition of glaucomatous LC changes in a patient. The present study therefore evaluated LC curvature in healthy subjects, as well as analyzing the factors associated with LC curvature.

## Results

This cross-sectional study initially involved 284 eyes of 142 Korean subjects. Seventeen subjects were excluded due to poor image quality, which prevented clear visualization of the anterior LC surface in at least two of the seven B-scan disc images. Thus, 250 eyes of 125 subjects were analyzed.

Table 1 summarizes the demographic characteristics of the included subjects. The 125 subjects included 79 women and 46 men, of mean age 49.02 ± 14.13 years. Intraocular pressures(IOP) at disc scanning, spherical equivalent, axial length (AXL), central corneal thickness (CCT), and mean deviation (MD) of visual field did not differ between the right and left eyes of the included subjects (*P* > 0.05 each).

**Table 1:** Demographic characteristics of the study subjects.

Variables	Participants (n = 125)	Right eyes (n = 125)	Left eyes (n = 125)	*P* value*	Correlation Coefficient (*P* value)†
Age, years	49.02 ± 14.13 (range, 20–83)				
Age distribution	125				
<40, *n* (%)	32 (25.6)				
41–50, *n* (%)	25 (20.0)				
51–60, *n* (%)	42 (33.6)				
≥61, *n* (%)	26 (20.8)				
Male/female	46/79				
Diabetes mellitus, *n* (%)	7 (5.6)				
Systemic hypertension, *n* (%)	20 (16.0)				
IOP at disc scanning, mmHg		12.18 ± 2.60	12.38 ± 2.56	0.273	**0**.**665 (**<**0**.**001)**
Spherical equivalent, D		−0.56 ± 1.76	−0.63 ± 1.75	0.178	**0**.**953 (**<**0**.**001)**
Axial length, mm		23.71 ± 1.06	23.71 ± 1.09	0.546	**0**.**937 (**<**0**.**001)**
Central corneal thickness, μm		555.00 ± 37.55	554.28 ± 37.51	0.718	**0**.**979 (**<**0**.**001)**
Visual field MD, dB		−0.37 ± 1.27	−0.32 ± 1.31	0.847	**0**.**956 (**<**0**.**001)**

IOP = intraocular pressure; D = diopters; MD = mean deviation; dB = decibel.

Data are reported as mean ± standard deviation or *n* (%), with statistically significant *P* values in boldface.

*Paired *t*-test: comparison of parameters between right and left eyes.

^†^Correlation coefficient: correlation of parameters in right and left eyes.

The 95% Bland-Altman limits of agreement between the measurements of lamina cribrosa curvature index (LCCI) by the two glaucoma specialists ranged from –1.23 to 1.37.

### LCCI Variation Between Subjects

**Figure 1:**
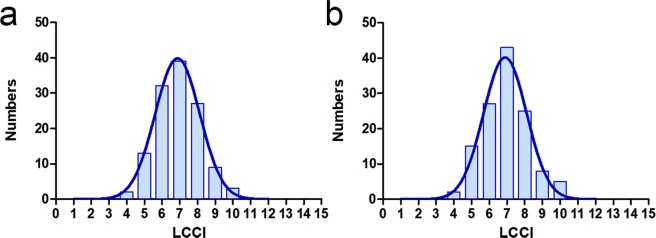
Histograms showing the distribution of LCCIs in the (**a**) right and (**b**) left eyes of the 125 healthy subjects. LCCI distribution in both eyes appeared as normal (Gaussian) curves (*P* = 0.200 by Kolmogorov-Smirnov test).

The mean LCCI (±standard deviation) was 7.46 ± 1.22. The mean frequency distribution of the LCCI showed Gaussian curves in both right and left eyes (*P* = 0.200, Fig. 1) ranging from 4.29 to 10.48.

**Figure 2:**
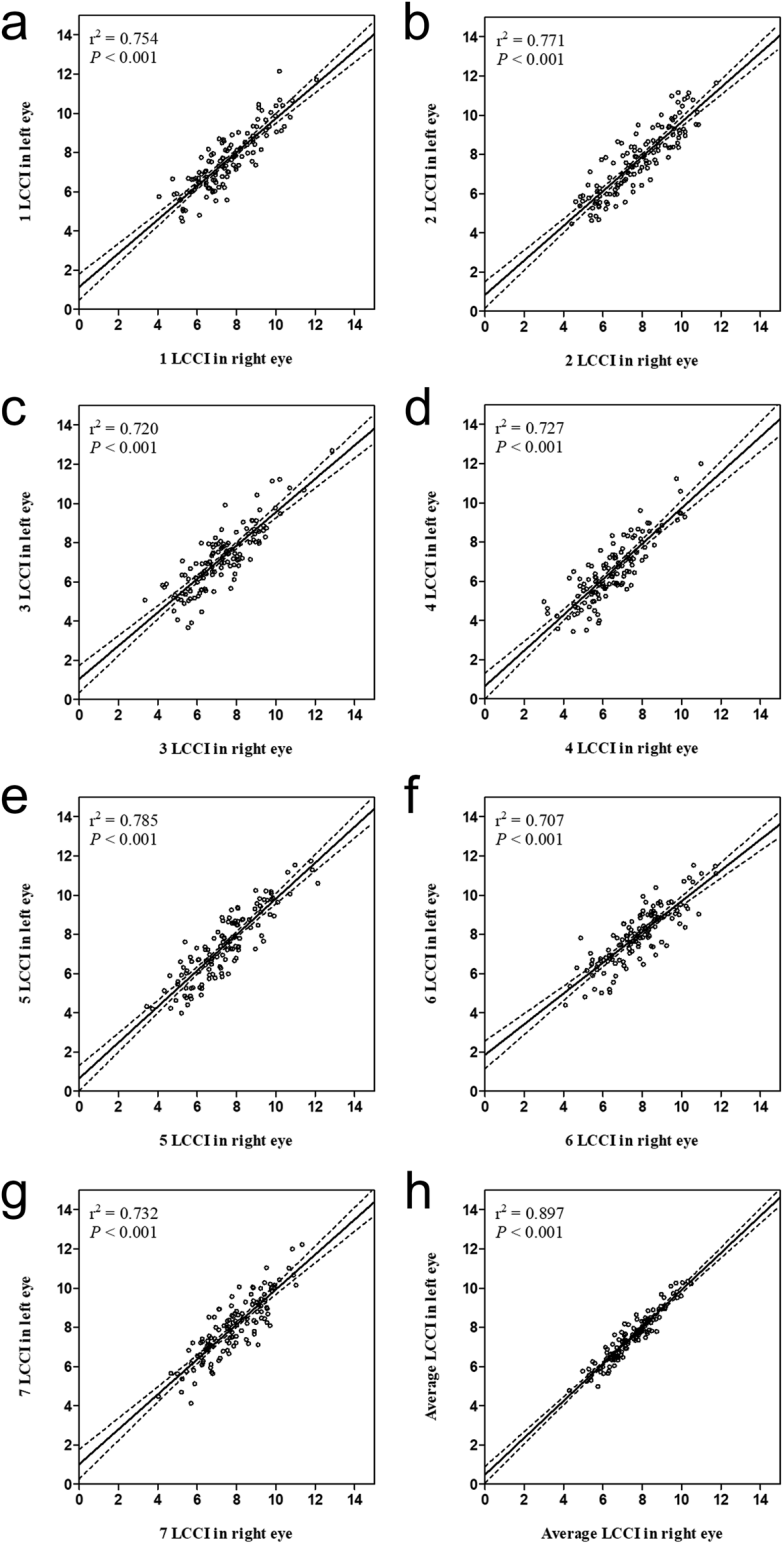
Scatterplots showing the relationship between the lamina cribrosa curvature index of right and left eyes at each location. *Solid lines* represent trend lines and *dotted lines* represent 95% confidence intervals.

There was no significant difference between the right (7.43 ± 1.23) and left eyes (7.49 ± 1.22). Rather, a strong inter-eye association was observed (correlation coefficient ≥0.841, all *P* < 0.001, Table 2, Fig. 2).

**Table 2:** LCCIs in the planes of right and left eyes in healthy subjects

Plane Number	All, *n* = 250	Right Eyes, *n* = 125	Left Eyes, *n* = 125	*P* value*	Correlation Coefficient (*P* value)†	Absolute Difference
1	7.58 ± 1.51 (4.06–12.15)	7.53 ± 1.53 (4.06–12.07)	7.62 ± 1.51 (4.50–12.15)	0.230	**0**.**868 (**<**0**.**001)**	0.08 ± 0.78
2	7.73 ± 1.69 (4.41–11.76)	7.77 ± 1.69 (4.41–11.76)	7.69 ± 1.69 (4.46–11.65)	0.269	**0**.**878 (**<**0**.**001)**	0.08 ± 0.83
3	7.21 ± 1.59 (3.34–12.86)	7.23 ± 1.59 (3.34–12.86)	7.20 ± 1.60 (3.67–12.70)	0.687	**0**.**848 (**<**0**.**001)**	0.03 ± 0.88
4	6.51 ± 1.57 (3.01–12.01)	6.48 ± 1.53 (3.01–10.99)	6.54 ± 1.63 (3.45–12.01)	0.451	**0**.**853 (**<**0**.**001)**	0.06 ± 0.86
5	7.41 ± 1.71 (3.44–12.14)	7.39 ± 1.69 (3.44–12.14)	7.43 ± 1.75 (3.99–11.74)	0.610	**0**.**886 (**<**0**.**001)**	0.04 ± 0.82
6	7.90 ± 1.51 (4.09–11.75)	7.82 ± 1.56 (4.09–11.75)	7.98 ± 1.45 (4.39–11.53)	0.030	**0**.**841 (**<**0**.**001)**	0.17 ± 0.85
7	7.87 ± 1.51 (4.03–12.23)	7.78 ± 1.47 (4.03–11.34)	7.96 ± 1.54 (4.13–12.23)	0.019	**0**.**856 (**<**0**.**001)**	0.17 ± 0.81
Average	7.46 ± 1.22 (4.29–10.48)	7.43 ± 1.23 (4.29–10.48)	7.49 ± 1.22 (4.79–10.35)	0.106	**0**.**947 (**<**0**.**001)**	0.06 ± 0.40

Data are reported as mean ± standard deviation (range).

*Paired *t*-test: comparison of parameters between right and left eyes.

^†^Correlation coefficient: correlation of parameters in right and left eyes.

Bonferroni correction was applied to raw data for measurements in the seven planes. Values significant after Bonferroni correction (*P* < 0.0071; 0.05/7) are shown in bold.

### LCCI Variation Within Eyes

**Figure 3:**
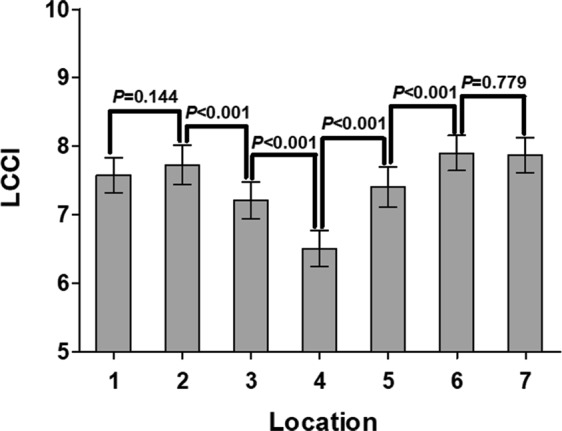
Average LCCIs in the seven individual planes of both eyes of the 125 included subjects. Some of the differences between planes were statistically significant. The LCCI was smallest at the mid-horizontal plane (plane 4).

The mean LCCI showed a significant variation between planes. The LCCI was larger in the superior and inferior peripheral regions than in the mid-horizontal plane, where the LCCI was the smallest (Fig. 3).

### Factors Associated with LCCI

Univariate analysis using a linear mixed model showed that axial length was significantly associated with LCCI in all planes (all *P* ≤ 0.003, Table 3, Supplementary Figure 1). Age was associated with LCCI at all planes (all *P* ≤ 0.006), except for planes 3 (*P* = 0.026) and 4 (*P* = 0.226). Multivariate analysis showed that only axial length was significantly associated with LCCI at planes 4 (*P* < 0.001), 5 (*P* = 0.001), and 6 (*P* < 0.001) and with average LCCI (*P* < 0.001, Table 4).

**Table 3:** Univariate linear mixed model analysis of factors associated with lamina cribrosa curvature index (*N* = 250 eyes).

Plane Number	Age Coefficient, *P* value	Gender Coefficient, *P* value	IOP Coefficient, *P* value	CCT Coefficient, *P* value	AXL Coefficient, *P* value	BMO width Coefficient, *P* value
1	**0**.**025**, ***P*** = **0**.**006**	0.322, *P* = 0.239	0.068, *P* = 0.017	0.000, *P* = 0.895	−**0**.**292**, ***P*** = **0**.**002**	0.0002, *P* = 0.585
2	**0**.**039**, ***P*** < **0**.**001**	0.384, *P* = 0.207	0.056, *P* = 0.069	0.002, *P* = 0.579	−**0**.**397**, ***P*** = **0**.**001**	−0.0002, *P* = 0.722
3	0.022, *P* = 0.026	0.104, *P* = 0.716	0.052, *P* = 0.096	0.002, *P* = 0.612	−**0**.**349**, ***P* = 0**.**003**	0.0002, *P* = 0.768
4	0.012, *P* = 0.226	0.231, *P* = 0.410	0.009, *P* = 0.763	0.001, *P* = 0.659	−**0**.**408**, ***P*** < **0**.**001**	0.0006, *P* = 0.275
5	**0**.**029**, ***P*** = **0**.**005**	−0.067, *P* = 0.829	−0.014, *P* = 0.642	−0.001, *P* = 0.777	−**0**.**520**, ***P*** < **0**.**001**	0.0005, *P* = 0.422
6	**0**.**029**, ***P*** = **0**.**001**	−0.160, *P* = 0.549	0.020, *P* = 0.514	0.005, *P* = 0.077	−**0**.**610**, ***P*** < **0**.**001**	−0.0004, *P* = 0.509
7	**0**.**027**, ***P*** < **0**.**001**	−0.278, *P* = 0.302	0.020, *P* = 0.497	0.001, *P* = 0.738	−**0**.**459**, ***P*** < **0**.**001**	0.0001, *P* = 0.574
Average	**0**.**028**, ***P*** = **0**.**001**	0.072, *P* = 0.749	0.018 *P* = 0.250	0.001, *P* = 0.672	−**0**.**388**, ***P*** < **0**.**001**	0.0002, *P* = 0.0.695

IOP = intraocular pressure; CCT = central corneal thickness; AXL = axial length, BMO = Bruch’s membrane opening.

Bonferroni correction was applied to raw data for measurements in the seven locations. Values significant after Bonferroni correction (*P* < 0.0071; 0.05/7) are shown in bold.

**Table 4:** Multivariate linear mixed model analysis of factors associated with lamina cribrosa curvature index (*N* = 250 eyes).

Plane	Age Coefficient, *P* value	Gender Coefficient, *P* value	IOP Coefficient, *P* value	CCT Coefficient, *P* value	AXL Coefficient, *P* value	IOP*CCT Coefficient, *P* value	BMO width Coefficient, *P* value
1	0.017 *P* = 0.104	0.543, *P* = 0.048	−0.129, *P* = 0.731	−0.005, *P* = 0.543	−0.273, *P* = 0.040	0.0004, *P* = 0.591	0.0002, *P* = 0.675
2	0.030, *P* = 0.008	0.704, *P* = 0.019	0.097, *P* = 0.809	0.002, *P* = 0.840	−0.327, *P* = 0.025	−0.0001, *P* = 0.931	−0.0004, *P* = 0.378
3	0.010, *P* = 0.346	0.360, *P* = 0.221	0.192, *P* = 0.644	0.003, *P* = 0.746	−0.344, *P* = 0.018	−0.0002, *P* = 0.743	−0.0001, *P* = 0.796
4	−0.007, *P* = 0.509	0.543, *P* = 0.054	0.288, *P* = 0.480	0.005, *P* = 0.559	−**0**.**524**, ***P*** < **0**.**001**	−0.0004, *P* = 0.501	0.0002, *P* = 0.674
5	0.009, *P* = 0.425	0.333, *P* = 0.285	−0.324, *P* = 0.422	−0.008, *P* = 0.371	−**0**.**506**, ***P*** = **0**.**001**	0.001, *P* = 0.426	0.0001, *P* = 0.851
6	0.007, *P* = 0.438	0.315, *P* = 0.201	−0.316, *P* = 0.421	−0.004, *P* = 0.658	−**0**.**603**, ***P*** < **0**.**001**	0.001, *P* = 0.380	−0.0005, *P* = 0.400
7	0.021, *P* = 0.038	0.024, *P* = 0.927	−0.374, *P* = 0.330	−0.007, *P* = 0.403	−0.325, *P* = 0.014	0.001, *P* = 0.299	0.0008, *P* = 0.299
Average	0.015, *P* = 0.063	0.378, *P* = 0.074	−0.052, *P* = 0.809	−0.001, *P* = 0.799	−**0**.**365**, ***P*** < **0**.**001**	0.0001, *P* = 0.718	−0.0001, *P* = 0.759

IOP = intraocular pressure; CCT = central corneal thickness; AXL = axial length, BMO = Bruch’s membrane opening.

IOP*CCT, product of IOP and CCT.

Bonferroni correction was applied to raw data for measurements in the seven locations. Values significant after Bonferroni correction (*P* < 0.0071; 0.05/7) are shown in bold.

## Discussion

Histologic examination has shown backward bowing of the LC in glaucomatous eyes^1,12^. In addition, displacement of the LC may be the earliest change in experimental glaucoma models^13–15^. These findings suggested that LC deformation is a principal event in glaucomatous optic neuropathy. Therefore, understanding variations of LC morphology in normal and glaucomatous eyes is important for diagnosing and managing glaucoma. Because LC deformation occurs as a posterior bowing^12,15^, evaluating LC curvature may be a useful in assessing LC morphology. Before applying LC curvature to clinical practice, however, it is essential to know the normal variations and factors associated with LC curvature. To our knowledge, this is the first study to examine variations in LCCI and factors related to LCCI in healthy subjects.

The present study analyzed ONH morphology using seven horizontal B-scan images. Although vertical or radial scans may also be used to evaluate the LC curvature, there are technical problems using these scans. LC has a bowtie-shaped horizontal central ridge on three-dimensional LC images^8^, with vertical scans showing that the LC has a “W-shape”. Therefore, LC curvature on vertical scans cannot be measured by a simple parameter such as LCCI. In addition, the LC curvature appears largely different according to the location or meridian of the scans when evaluated using vertical or radial scans (Supplementary Figure 2 and Video 1). Even in glaucomatous eyes in which the LC is largely deformed, the horizontal scan shows a relatively uniform shape with different degree of curvature depending on the location of scan (Supplementary Video 2).

We observed a significant negative correlation between axial length and LCCI. There are three possible explanations about this finding. First, this,which may be due to deformation of the optic disc during axial elongation. As the eyeball becomes longer in the axial direction, the temporal sclera moves backward and becomes flatter^16^, pulling the optic nerve in the temporal direction. As a result, the tissue including the connecting portion of the oblique disc and the sclera becomes flatter. This may result in eyes of longer axial length having a smaller LCCI. Second, the overhanging scleral flange in an oblique disc changes the location of the LCCI peripheral point, such that it is closer to the center of the LC. This could result in LCCI being smaller in myopic eyes or those with tilted discs/oblique optic nerve insertions. Third, it is also plausible that the thinner and more compliant sclera in myopic eyes is more prone to scleral canal expansion, thereby pulling the LC forward (flatter) in the canal and resulting in a smaller LCCI in more myopic eyes. In the present study, eyes with tilted or torted discs were excluded. If they were included, the negative relationship between LCCI and axial length would have been more pronounced. The significant association of axial length with the LCCI indicates that AXL should be considered when evaluating LCCI.

Age showed a significant positive relationship with LCCI on univariate, but not on multivariate analysis. This result may be due to a strong negative correlation between age and axial length (Pearson’s correlation coefficient −0.523, *P* < 0.001, n = 250, data not shown) as previously reported^17^.

We have reported that LCCI is reduced when IOP is lowered in patients with glaucoma^18^, indicating that changes in LC curvature are dependent on IOP. In contrast, the present study showed no correlation between LCCI and IOP. This was likely due to the inclusion in the present study of only healthy subjects with IOP ≤ 21 mmHg. These findings indicate that IOP within a normal range does not significantly influence LC curvature in healthy subjects.

LCCI was not zero in healthy eyes, which is consistent with our previous study^11^. This curvature can indicate the acute position of the LC at the moment of the scan, or change over time with age. We consider that the former is not likely the case. This idea is based on the finding that the LC position did not instantly change by acute IOP elevation^19^. The observed LCCI in our healthy subjects may be the sum of the innate curve of the LC and the accumulated change over time derived from the translaminar pressure difference.

The ONH showed that LCCI was smallest in the mid-horizontal region. This finding is in agreement with prior imaging study showing a horizontal central ridge in the LC and a humplike structure in the center of the ridge^8^. In contrast, the superior and inferior regions with large LC curvature are consistent with the regional differences in susceptibility to glaucomatous damage^20^.

This study had several limitations. First, all subjects included were Korean. Further study is needed in other ethnic groups. Second, assessment of LC curvature from the LC insertion points would have allowed the precise quantification of LC configuration. However, it is not possible to identify LC insertion points in all eyes. LCs outside the BMO cannot be visualized with the currently available EDI SD-OCT due to overlying large vessels or rim shadowing. However, according to our previous study, there was no significant difference between LCCI measured from the whole LC and that measured on the LC within BMO in eyes where the entire LC (i.e., between its insertions) was visible^18^. Therefore, LCCI measured within BMO may surrogate the curvature of entire LC. Third, eyes with discs with deformed morphology, such as tilted or torted, were not included in this study. Therefore, the normative data shown in this study cannot be applied to those eyes.

In conclusion, this study presents the normal profiles of LCCI. The current data may enable the clinical application of LCCI for detecting and managing glaucoma.

## Methods

This investigation was based on an ongoing prospective study, Investigating Glaucoma Progression Study (IGPS), being performed at the Seoul National University Bundang Hospital Glaucoma Clinic^21,22^. Subjects with healthy eyes enrolled in the IPGS were selected for the present study. This study was approved by the Seoul National University Bundang Hospital Institutional Review Board and followed the tenets of the Declaration of Helsinki. All subjects provided written informed consent.

### Study Subjects

Each subject enrolled in the IGPS underwent comprehensive ophthalmic examinations including visual acuity measurements, Goldmann applanation tonometry, refraction tests, slit-lamp biomicroscopy, gonioscopy, dilated stereoscopic examination of the optic disc, stereo disc photography (EOS D60 digital camera, Canon, Utsunomiya-shi, Tochigiken, Japan), spectral-domain optical coherence tomography (SD-OCT, Spectralis OCT, Heidelberg, Engineering, Heidelberg, Germany), measurements of CCT (Orbscan II, Bausch & Lomb Surgical, Rochester, NY, USA), corneal curvature (KR-1800; Topcon, Tokyo, Japan), AXL (IOL Master version 5, Carl Zeiss Meditec, Dublin, CA, USA), and standard automated perimetry (Humphrey Field Analyzer II 750, 24-2 Swedish interactive threshold algorithm, Carl Zeiss Meditec).

Both eyes of healthy subjects had an IOP ≤ 21 mmHg with no history of increased IOP, an absence of a glaucomatous disc appearance, no visible retinal nerve fiber layer (RNFL) defect on red-free photography, and a normal visual field. Absence of a glaucomatous disc appearance was defined as an intact neuroretinal rim without peripapillary hemorrhages, notches, or localized pallor. A normal visual field was defined as the absence of glaucomatous visual field defects and neurologic field defects. A glaucomatous visual field defect was defined as (1) outside the normal limits on glaucoma hemifield test; (2) three abnormal points with *P* less than 5% probability of being normal, including one with *P* less than 1% by pattern deviation; or (3) a pattern standard deviation less than 5%, confirmed on two consecutive tests. Visual field measurements were considered reliable when false-positive/negative results were less than 25% and fixation losses were less than 20%.

Eyes included in the present study were required to have a best-corrected visual acuity of at least 20/40, spherical refraction within −6.0 to +3.0 diopters (D), and cylinder correction within −3.0 to +3.0 D without a tilted appearance (defined as a tilt ratio of the longest to the shortest diameters of the optic disc >1.3)^23,24^ or torsion of the optic disc (defined as a torsion angle [the deviation of the long axis of the optic disc from the vertical meridian] of >15°)^24,25^, because LC may be distorted in these eyes. Subjects with a history of intraocular surgery other than cataract extraction, intraocular disease (e.g., diabetic retinopathy, retinal vein occlusion, or optic neuropathies), or neurologic disease (e.g., pituitary tumor) that could cause visual field loss were excluded. Eyes were also excluded when a good-quality image (i.e., quality score > 15) could not be obtained due to media opacity or lack of patient cooperation. Subjects were also excluded when images did not allow clear delineation of the anterior border of the central LC. Both eyes of each subject were evaluated to assess LCCI symmetry.

The IOP at disc scanning was defined as the IOP at the time of obtaining SD-OCT images.

### Enhanced Depth Imaging OCT of the Optic Nerve Head

The optic nerve was imaged using the enhanced-depth-imaging technique of the Spectralis OCT system. The details and advantages of this technology in evaluating the LC have been described previously^26,27^. In brief, imaging was performed through undilated pupils using a rectangle subtending 10° × 15° of the optic disc. This rectangle was scanned with approximately 75 B-scan section images separated by 30 to 34 μm, with the scan line distance determined automatically by the machine. Approximately 42 SD-OCT frames per section were averaged. Using Spectralis OCT, the images were obtained only when the quality score is higher than 15. This protocol provided the best trade-off between image quality and patient cooperation^28^. Potential magnification errors were avoided by entering the corneal curvature of each eye into the Spectralis OCT system before scanning.

Three-dimensional (3D) volumetric images were reconstructed from the B-scan images and en face images were constructed from the 3D images using image-processing software (Amira 5.2.2; Visage Imaging, Berlin, Germany).

### Quantification of Posterior Bowing of the LC

**Figure 4:**
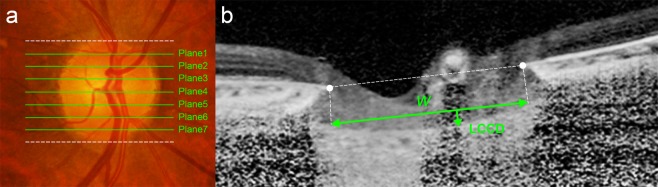
Measurement of the lamina cribrosa curvature index (LCCI). (**a**) Seven horizontal *green solid lines* shown on the disc photography indicate the locations where the LCCI was measured. *White dotted lines* indicate upper and lower disc margins, separately. (**b**) Spectral-domain optical coherence tomography (SD-OCT) B-scan image obtained in plane 2 as shown in (**a**). (**b**) The LCCI was measured by dividing the lamina cribrosa curve depth (LCCD) by the width of the anterior LC surface reference line (*W*) and multiplying by 100.

After the 3D image was reconstructed, seven B-scan images that divided the optic disc diameter into eight equal parts vertically were selected for each eye. These seven B-scan lines (green lines) were defined as planes 1 to 7, representing superior to inferior regions (Fig. 4). In this model, plane 4 corresponds to the mid-horizontal plane, and planes 2 and 6 correspond approximately to the superior and inferior mid-periphery, respectively.

To assess the posterior bowing of the LC, the LCCI was defined as the inflection of a curve representing a section of the LC, as described^11,27^. Briefly, the LC surface reference line was set in each B-scan by connecting the two points on the anterior LC surface that met the lines drawn from each Bruch’s membrane termination point perpendicularly to the BMO reference line. The length of this reference line was defined as the width (*W*). The lamina cribrosa curve depth (LCCD) was defined as the maximum depth from this reference line to the anterior LC surface, was measured, and LCCI was calculated as (LCCD/*W*) × 100.

To measure the LCCI for each plane, each B-scan image was enlarged on the computer screen so that each pixel was clearly visible when the caliper tool was used. Two experienced observers (SHL and EJL), who were masked to the clinical information measured the LCCI, and the measurements by the two observers were averaged for the analysis. The average LCCI for each eye was calculated as the mean measurements at the seven points of the LC.

### Statistical Analysis

The Bland-Altman limits of agreement were used to measure the inter-observer reproducibility of measurements of the LCCI. Inter-eye comparisons of LCCI within subjects were analyzed using paired *t*-tests, and the correlation between right and left eyes within subjects were assessed by Pearson correlation analysis. The raw data for *t*-tests were subjected to Bonferroni’s correction, based on the number of comparisons within each analysis. A linear mixed model was used to assess the association of clinical factors with the LCCI (univariate and multivariate), including both eyes of each subject. For multivariate analysis, the interaction term (CCT × IOP) was also included. *P* values less than 0.05 were regarded as statistically significant. All statistical analyses were performed using the Statistical Package for Social Sciences (version 22.0, SPSS, Chicago, IL, USA).

## Supplementary information

Supplementary Information

S1

S2

## Data Availability

Data supporting the findings of the current study are available from the corresponding author on reasonable request.
