# Supplementary material for: Lamina Cribrosa Curvature in Healthy Korean Eyes
Source: Sci Rep. 2019 Feb 11;9:1756. doi: 10.1038/s41598-018-38331-7 (PMC6370828; doi:10.1038/s41598-018-38331-7)
Supplement: Supplementary file 1 — Supplementary Information [file 41598_2018_38331_MOESM1_ESM.pdf]

Supplementary Information

Lamina Cribrosa Curvature in Healthy Korean Eyes

Seung Hyen Lee,[1] Tae-Woo Kim,[2*] Eun Ji Lee,[2] Michaël J. A. Girard,[3, 4] Jean Martial Mari[5]

[1]Department of Ophthalmology, Bundang Jesaeng General Hospital, Daejin Medical Center, Seongnam, Korea

[2]Department of Ophthalmology, Seoul National University College of Medicine, Seoul National University Bundang Hospital, Seongnam, Korea

[3]Department of Biomedical Engineering, National University of Singapore, Singapore

[4]Singapore Eye Research Institute, Singapore National Eye Centre, Singapore

[5]Université de la Polynésie française, Tahiti, French Polynesia

Supported by grant no. 02-2016-023 from the Seoul National University Bundang Hospital Research Fund. The funding organization played no role in the design or conduct of this research. The other authors have no proprietary or commercial interests in any of the materials discussed in this article.

Corresponding author:

Tae-Woo Kim, MD.

Department of Ophthalmology, Seoul National University College of Medicine, Seoul National University Bundang Hospital, 300 Gumi-dong, Bundang-gu, Seongnam, Gyeonggi-do 13620, Korea

Tel: 82-31-787-7374

Fax: 82-31-787-4057

E-mail: twkim7@snu.ac.kr

Supplementary information file includes two videos and two figures (Video S1, S2 and Figure S1, S2).

Supplementary Video legends

Supplementary Video S1

Radial scans of the optic nerve head. Note that the LC configuration in each meridian is remarkably irregular.

Supplementary Video S2

Raster scans of the optic nerve head. Note that the LC configuration is relatively regular along the planes with a flat or U shaped appearance with differing regional steepness.

Supplementary Figure legends

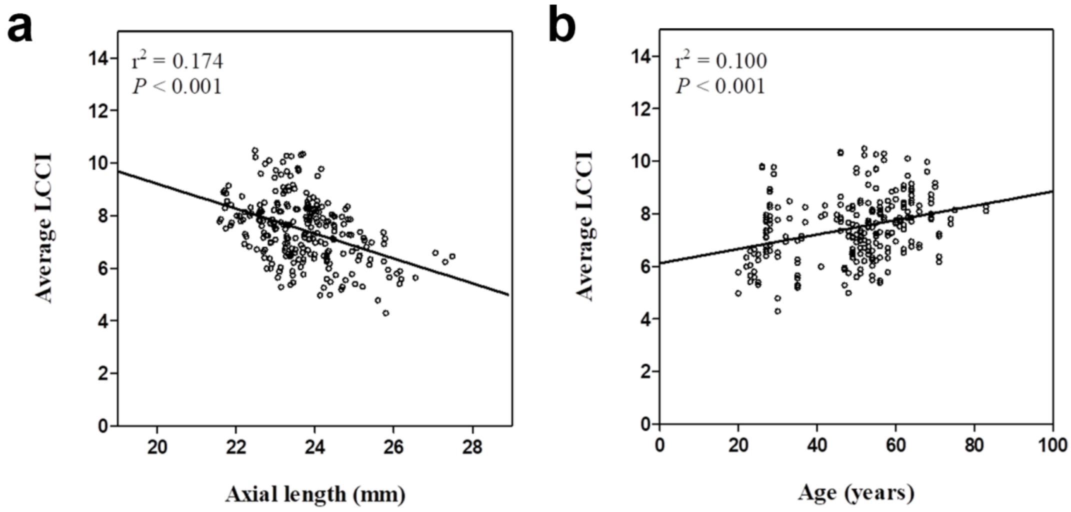

Supplementary Figure S 1. Scatterplots for axial length (a) and age (b) associated with average LCCI. *Solid lines* represent trend lines.

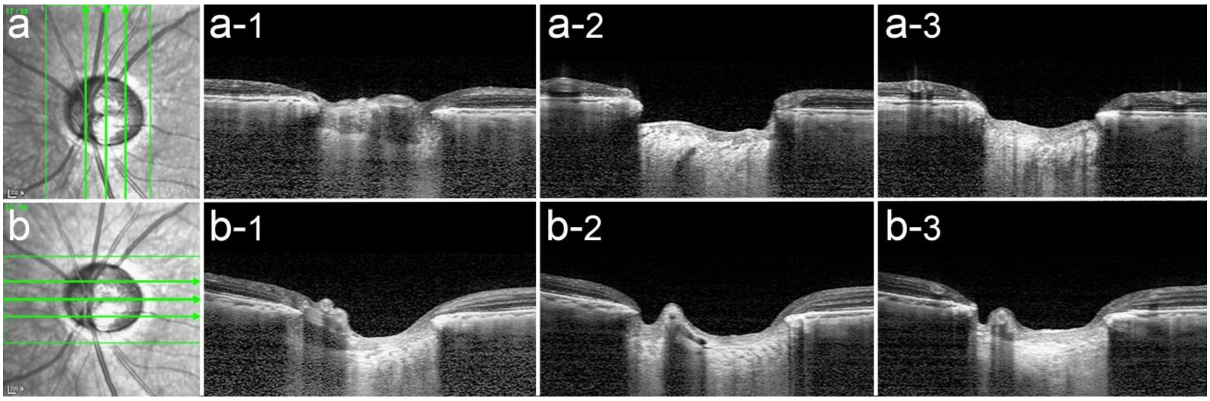

Supplementary Figure S 2. Comparison of (a) vertical and (b) horizontal scans. It is difficult to construct the image of the LC deformation in ones' mind by combining the 3 vertical scans particularly in the inferior region in this case (a). In contrast, one can easily surmise the LC morphology using horizontal scans (b).
